# Abdominal Ultrasound and Its Diagnostic Accuracy in Diagnosing Acute Appendicitis: A Meta-Analysis

**DOI:** 10.3389/fsurg.2021.707160

**Published:** 2021-06-28

**Authors:** Jian Fu, Xu Zhou, Liang Chen, Sheng Lu

**Affiliations:** Department of Ultrasound, Affiliated Haian Hospital of Nantong University, Nantong, China

**Keywords:** acute appendicitis, ultrasound, histopathology, computed tomography (CT scan), emergency department

## Abstract

**Background:** Acute appendicitis (AA) is a common cause of abdominal pain encountering unnecessary surgeries in emergency departments. The present meta-analysis aims to assess the accuracy of abdominal ultrasound in suspected acute appendicitis cases in terms of sensitivity, specificity, and post-test odds for positive and negative results.

**Materials and Methods:** An extensive and systematic search was conducted in Medline (*via* PubMed), Cinahl (*via* Ebsco), Scopus, and Web of Sciences from 2010 till the end of March 2021. Two authors analyzed studies for inclusion, collected results, and conducted analyses separately. Examination of the histopathological tissue collected during appendectomy served as a gold standard for determining the final diagnosis of appendicitis. The accuracy was determined by evaluating sensitivity, specificity, positive predictive value (PPV), negative predictive value (NPV), diagnostic odds ratio.

**Results:** Out of 3,193 references, a total of 18 studies were selected. Overall sensitivity of 77.2% (95% CI – 75.4–78.9%) and specificity of 60% (95% CI – 58–62%) were observed. The diagnostic odds ratio of 6.88(95% CI 1.99–23.82) was obtained.

**Conclusion:** Abdominal ultrasound shows significant accuracy of diagnosis in patients with suspected acute appendicitis.

## Introduction

Acute appendicitis (AA) is considered one of the most common causes of surgical emergencies worldwide (1). The reported mortality rate is from <1% in younger patients up to 5% in the elderly (2, 3). Abdominal pain is one of the most common cause of acute appendicitis, yet 34% of cases (4, 5) are still misdiagnosed, which results in unnecessary surgery. This high rate of negative appendectomy can be decreased by careful and accurate diagnosis of appendicitis, thus preventing acute appendicitis from progressing to perforation and peritonitis (6).

Abdominal ultrasound (US), computed tomography (CT), and magnetic resonance imaging (MRI) have also been used in the identification or exclusion of AA. The US's sensitivity and specificity in identifying AA have been reported to range from 71 to 92% and 83%, respectively, for normal contrast-enhanced CT 98 and 91%, and MRI 97 and 93% (7–9).

Computed Tomography (CT) is the most preferred diagnostic imaging modality to rule out AA in the adult population. Although its accuracy is high, with sensitivities ranging from 90 to 96% and specificities ranging from 94 to 98%; however, there are certain limitations, including radiation exposure, risk of contrast administration, increased resource utilization, high cost (7, 8), and development of future malignancies (9). However, to eliminate such constraints; the incidence of negative appendicectomy rate, and perforation, clinicians often go for imaging modalities such as abdominal ultrasound (US) as an alternative diagnostic approach because it is easy, inexpensive method, easily portable, and has high precision (10) in cases of suspected appendicitis both in children and adults.

CT or US did not improve the diagnostic precision of AA (3). Despite its confirmed low diagnostic accuracy, the US has been listed as a potential method for diagnosing AA because it does not require radiation. However, despite being a non-ionizing process, the question remains whether the US can contribute to the management of patients with AA suspicion without causing further management delays. Patients with stomach pain who do not have AA are exposed to invasive surgery if the condition is misdiagnosed. It can happen in up to 34% of cases (4, 5).

### Rationale

When patients with AA are misdiagnosed as not having the condition, a mandatory appendectomy may be postponed, and severe complications may occur, with a mortality rate of about 1.5% (2). Legal charges against both non-surgical and surgical subspecialties have been identified in delayed or incorrect diagnosis leading to adverse effects. As a result, it is essential to correctly identify AA in patients who exhibit symptoms and signs suggestive of the condition.

### Objective

The present study is an approach to correlate the diagnostic accuracy of abdominal ultrasound to histopathology, which is considered a gold standard in acute appendicitis (AA) cases in terms of sensitivity and specificity for positive and negative US results.

## Materials and Methods

We followed the Preferred Reporting Items for Systematic Reviews and Meta-Analyses (PRISMA) normative recommendations in this study with the registration number NU#/IRB/2020/1022.

### Search Strategy

The present meta-analysis is an extensive search conducted in Medline (*via* PubMed), Cinahl (*via* Ebsco), Scopus, Web of Sciences, from 2010 till the end of March 2021. The search was performed based on the keywords related to diagnostic accuracy, abdominal ultrasound, acute appendicitis, diagnosis, decreased CT use, and ultrasonography. All articles selected were based on PRISMA guidelines. The selection was irrespective of language or publication status or whether the study conducted was done prospectively or retrospectively. Table 1 summarizes the demographic details of the studies included from the search query of the Medline database with the considered variables. The primary focus of the present study was to assess the efficacy of ultrasound for cases of acute appendicitis in all age groups. To rule out the effectiveness of ultrasound in cases of acute appendicitis; sensitivity, specificity, positive predictive value (PPV), negative predictive value (npv), and diagnostic odds ratio's were assessed with the help of true positive (TP), false positive (FP), true negative (TN), false negative (FN) values.

**Table 1:** Demographic summary of included studies with ultrasound and histopathology in suspected cases of acute appendicitis.

**References**	**Study type**	**Study duration**	**Total Sample size**	**Age/mean age**	**Gender M/F**	**Sonographer**	**Type of US probe**	**Ultrasonography performed**	**Compared with histopathologic (H/P) correlation**	**Other methods for correlation used**
Samudra et al. (11)	Prospective comparative study	2 years	200	12–60 yrs	Unclear	Not stated	Not stated	200	H/P 127/200 (63.5%)	Modified Alvarado score
Austin-Page et al. (12)	Retrospective study	5 years	267	1–18 yrs	135/132	Not stated	Not stated	267	H/P done	CT
Farooq et al. (13)	Cross-sectional study	10 months	200	9–55 yrs	117/83	senior resident	Toshiba Aplio 500 machine, using both curvilinear and linear probes.	200	175/200	Alvarado Score
Crocker et al. (14)	Retrospective study	2 yrs	798	32.7 ± 16	221/557	Technician/ radiology residents	(iU22, Philips Healthcare) using 5-MHz curvilinear and 9- and 12-MHz linear transducers	562	H/P done	STARD guidelines
Dhatt S et al. (15)	Retrospective study	1 yr 5 months	134	3–18 yrs	69/65	radiology residents	Not mentioned	89	Surgery and H/P	Alvarado Score
Khan U. et al. (16)	Prospective study	3 years 5 months	223	3–14 yrs	143/80	senior technicians	Not mentioned	223	H/P 215/223	CT
Pedram et al. (10)	Cross-sectional study	Not mentioned	230	5–15 yrs	109/121	Not mentioned	Not mentioned	230	H/P 150/230	–
Mirza et al. (17)	Retrospective study	Not mentioned	1,115	2–16 years	714/401	Senior radiologists	Toshiba Xario (Toshiba Medical Systems Corporation, Japan) with 3.5-10 MHz probes.	1,115	H/P done 358/1,115	CT
Tyler et al. (18)	Retrospective cohort study	2 years 3 months	174	13–59 yrs	27/147	Radiologist	Philips IU22 linear 28-Hz probe (Philips Healthcare, Andover, MA)	174	H/P 25/174	CT
Shahbazipar et al. (19)	Cross-sectional study	Not mentioned	121	18.2–88.7 years	67/54	Radiologist and emergency medicine	Sonoace X8, Medison (Medison Company, Seoul, South Korea). The linear 7.5 MHz US probe was used.	121	H/P 46/121	Surgery
Karimi et al. (20)	Prospective study	1 year	108	mean age of 23.91 ±7.46	61.1% male	Senior radiologists	Ultrasonography machine (HS2000, Honda, Korea) with a linear probe and 5–7.5 MHz frequency.	108	H/P done	CT
Hussain et al. (21)	Cross-sectional study	7 months	60	10–70 yrs	48/12	radiologist	Toshiba Aplio and GE Logic 500 Pro Series machines using a 3.5/5.0 MHz convex-array transducer	60	H/P done 34/60	Color Doppler USG
Parsijani et al. (22)	retrospective cross-sectional study	1 year	238	4–76 yrs	160/78	radiology residents	Not mentioned	128	H/P done 98/128	Alvarado score
Sezer et al. (23)	Retrospective study	Not mentioned	91	18–54 yrs	44/47	Not mentioned	Toshiba Folio 8 brand machine with 3.75 and 8 MHz linear probes using the Puylaert's gradual press technique	91	77/91	–
Burford et al. (24)	Prospective study	Unclear	54	3–16 years	28/26	Surgical resident	Sonosite Micromax (Seattle, WA,) US with a 6- to 13-MHz high-frequency linear transducer	54	H/P 29/54	U/S combined with physical examination & history taking
Gokce et al. (25)	Prospective study	1 year	235	Mean 28 yrs	190/45	Not mentioned	Linear probe	235	150/235	–
Peixoto et al. (26)	Prospective study	Not mentioned	156	>12 yrs	82/74	Radiologist	Philips HDI-4000 with transducers of low and high frequency.	156	131/156	
Memisoglu et al. (27)	Retrospective study	1 year	196	7–81 yrs	122/74	Radiologist	USG (Siemens Sonoline G50) with a 3.5 MHz convex and 7.5 MHz linear probe	196	162/196	–

It did not matter whether the data was compiled prospectively or retrospectively or in what language it was written. Two authors (JF and XZ) separately scanned the sources for related studies. For publications that at least one of the writers thought was significant, full texts of the sources were collected. To further exclude obsolete references, complete texts were obtained. Abstracts were only used if they included enough information for the study. Two researchers (LC and SL) independently collected data from included research.

### Inclusion and Exclusion Criteria

The studies evaluated the diagnostic accuracy of ultrasound for acute appendicitis in all age groups from 2010 to 2021. The histopathology report for the same was defined as the reference standard included in the present study. Only full-text data were included in the present study. Exclusion criteria included insufficient data, reference standard other than histopathology report, any relevant studies but published before 2010, and studies including pregnant females suffering from acute appendicitis to reduce the chances of risk of bias and heterogeneity.

### Evaluation of the Analytical Standard

The quality assessment of diagnostic accuracy tests assessment tool (QUADAS-2) (28) was used to determine the methodological quality of the included studies. The methodological validity of the included studies was evaluated by two reviewers (JF and XZ) separately. SL was in charge of resolving any disagreements between authors.

## Statistical Analysis

A 2 × 2 table was made, based on which pooled sensitivity, specificity, and diagnostic odds ratio were calculated employing the DerSimonian Lair technique. The diagnostic odds ratio was also evaluated with a higher DOR value indicating better diagnostic accuracy of the test. The Cochran Q statistic and I (2) index evaluated the heterogeneity of the studies included. Meta disc software was used for the creation of forest plots. We also presented the data obtained from the various studies in the form of summary points of sensitivity and specificity in receiver operating characteristics (SROC) space with corresponding 95% confidence regions created using Review Manager 5 (29).

### Analysis of Sensitivity

Excluding participants with ambiguous findings can lead to an overestimation of diagnostic test accuracy. As a result, we conducted a sensitivity study in which we incorporated uninterpretable results in the analysis and evaluated diagnostic precision. All uninterpretable results were considered incorrect, comparing the outcomes to those of the principal analysis, which removed uninterpretable results.

### Investigation of Sources of Heterogeneity

We used metaregression to investigate heterogeneity in the included experiments, introducing various sources of heterogeneity as covariates and fitting a bivariate model. We used the probability ratio test to see whether a covariate has a significant impact on the description sensitivity and precision. For any of the subgroups, a *p*-value of 0.05 was found statistically significant. Full-text publication vs. abstracts, high vs. low risk of bias in included studies, prospective vs. retrospective studies, studies that included only adults vs. those that included mixed adult and pediatric populations, proportion of female participants, proportion of obese patients, type of ultrasound probe, and ultrasonographer experience were among the sources of heterogeneity that we investigated.

## Results

### Literature Search Results

**Figure 1:**
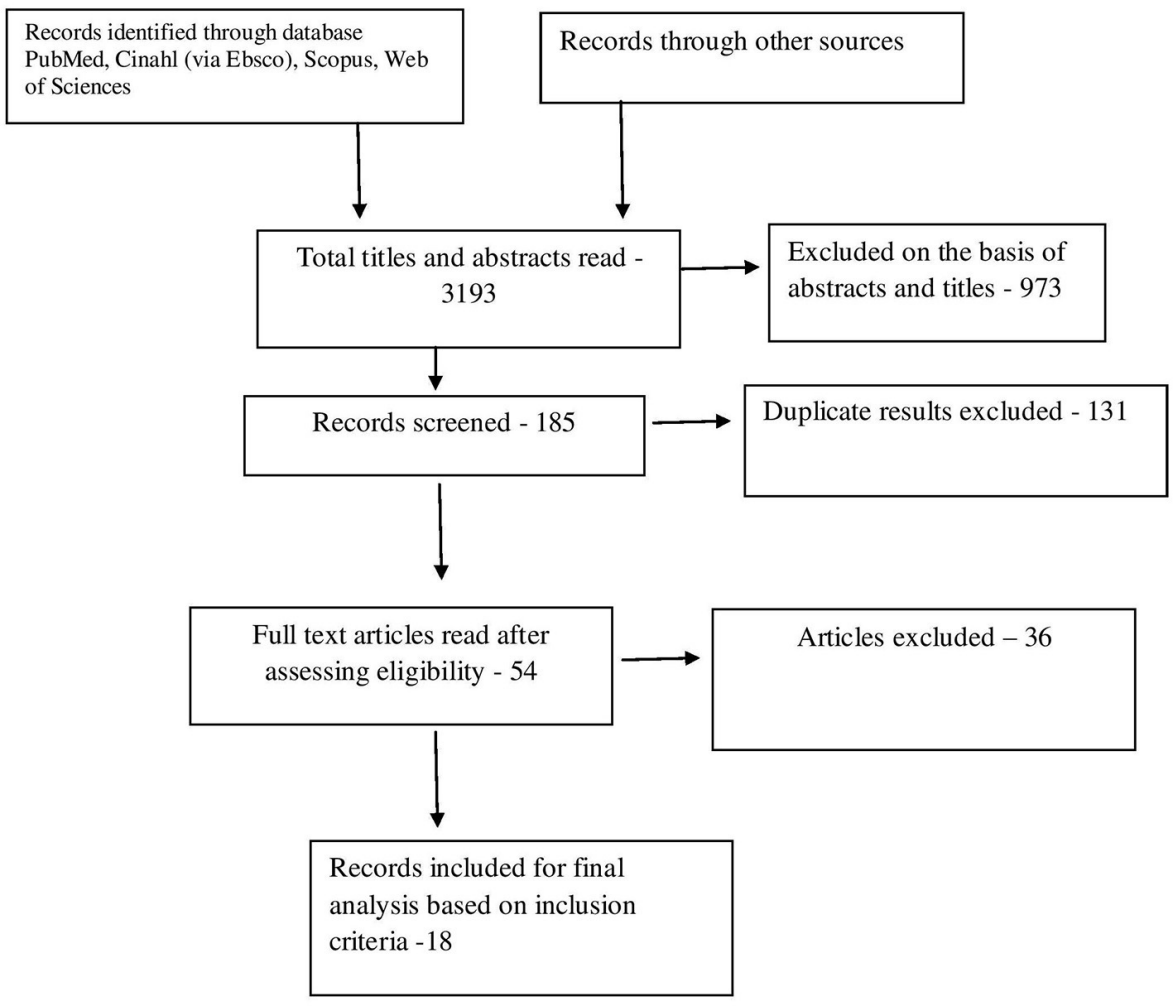
Flow chart diagram for article inclusion based on PRISMA guidelines.

Through electronic scans, we found a total of 3,193 studies. By reading titles and abstracts, we excluded 973 on reading titles and abstracts and 2,035 invalid references. Out of 185 studies, around 131 studies were excluded based on duplicity. Full-text publications were required for final screening was 54 out of which 36 were excluded based on the inclusion criteria. The research and meta-analysis contained 18 studies that met the inclusion criteria, i.e., based on the accuracy of abdominal ultrasound for acute cases of appendicitis, as shown in Figure 1. Inappropriate comparison criteria and inadequate evidence to create 2 × 2 tables for review were the key reasons for the omission.

Table 1 shows the demographic details of the studies included in the present meta-analysis describing study author, year of publishing, study type, study duration, total sample size, age, gender, details of the sonographer, type of US probe used, the sample size in which ultrasound was conducted, histopathology report which was considered as the gold standard for the comparison and any other method of correlation used for diagnosis. A total of 4,209 patients were included in the present meta-analysis. All studies were released as full-text papers, six of which were prospective, eight of which were retrospective, and four were cross-sectional. The participants' age ranged from 14 to 60 years old, and the majority of research did provide information about the operator's background (13 out of 18) or the kind of US probe used (12 out of 18).

### Risk of Bias Assessment

Individual reports' estimated sensitivity ranged from 75.4 to 78.9%, and specificity from 58 to 62%. Thus, according to the QUADAS-2 tool, all included experiments had a low chance of bias (Table 2).

**Table 2:** Risk of bias assessment.

**References**	**Patient selection**	**Index test**	**Reference standard**	**Flow and timing**	**Patient selection**	**Index test**	**Reference standard**
Samudra et al. (11)	L	L	L	L	L	L	L
Austin-Page et al. (12)	L	L	L	L	L	H	L
Farooq et al. (13)	L	L	L	U	L	L	L
Crocker et al. (14)	L	L	L	L	L	L	L
Dhatt et al. (15)	L	L	H	L	L	L	L
Khan et al. (16)	L	L	L	L	L	L	L
Pedram et al. (10)	L	L	L	L	L	L	L
Mirza et al. (17)	L	L	L	L	L	L	L
Tyler et al. (18)	L	L	H	L	L	L	L
Shahbazipar et al. (19)	L	L	L	U	L	L	L
Karimi et al. (20)	H	L	L	L	L	L	L
Hussain et al. (21)	H	L	L	L	L	L	L
Parsijani et al. (22)	L	L	L	L	L	L	L
Sezer et al. (23)	L	L	L	L	L	L	L
Burford et al. (24)	L	L	L	L	L	L	L
Gokce et al. (25)	L	L	L	L	L	L	L
Peixoto et al. (26)	L	L	L	L	L	L	L
Memisoglu et al. (27)	L	L	L	L	L	L	L

### Meta-Analysis Results

**Figure 2:**
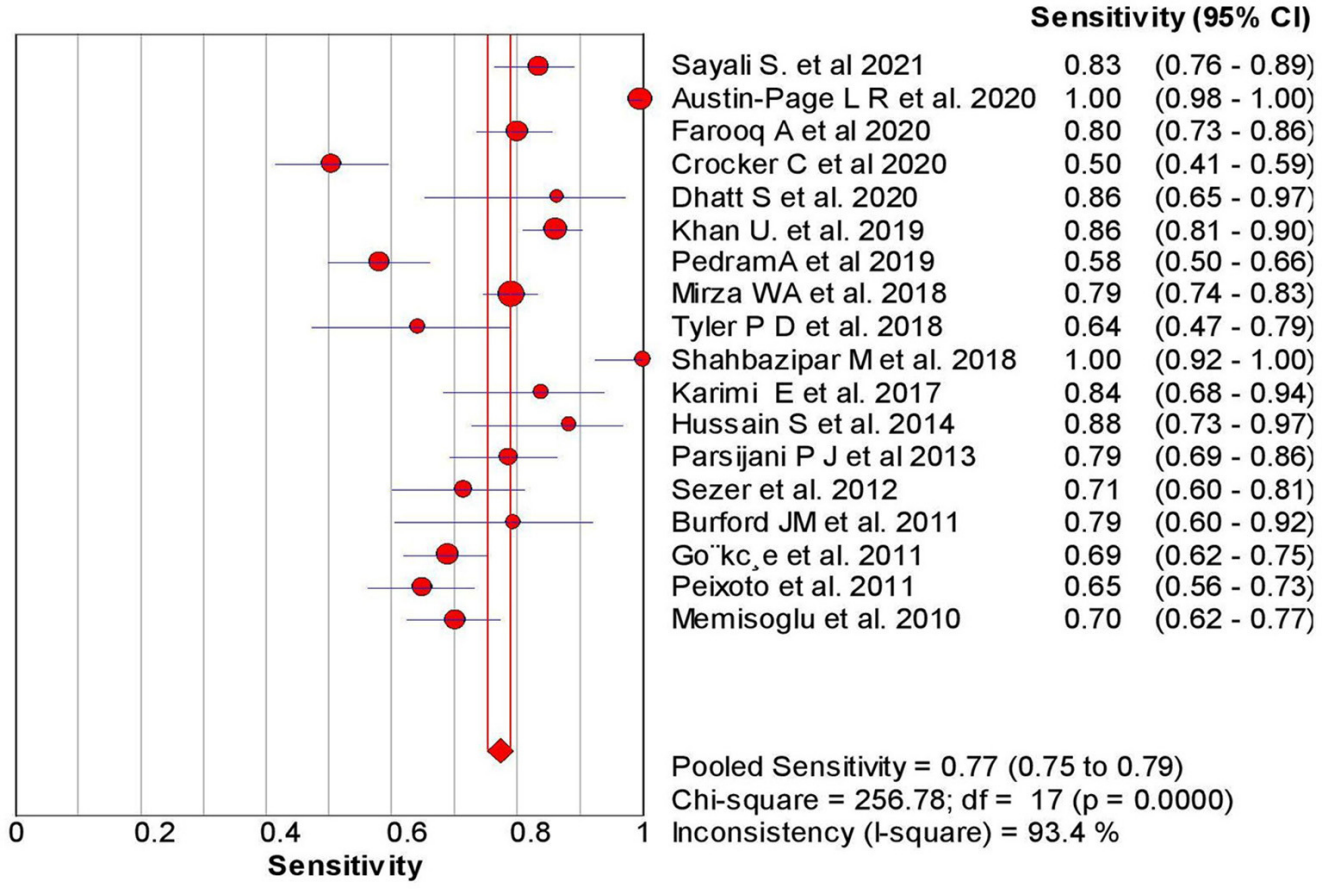
Sensitivity of ultrasound in acute appendicitis cases.

**Figure 3:**
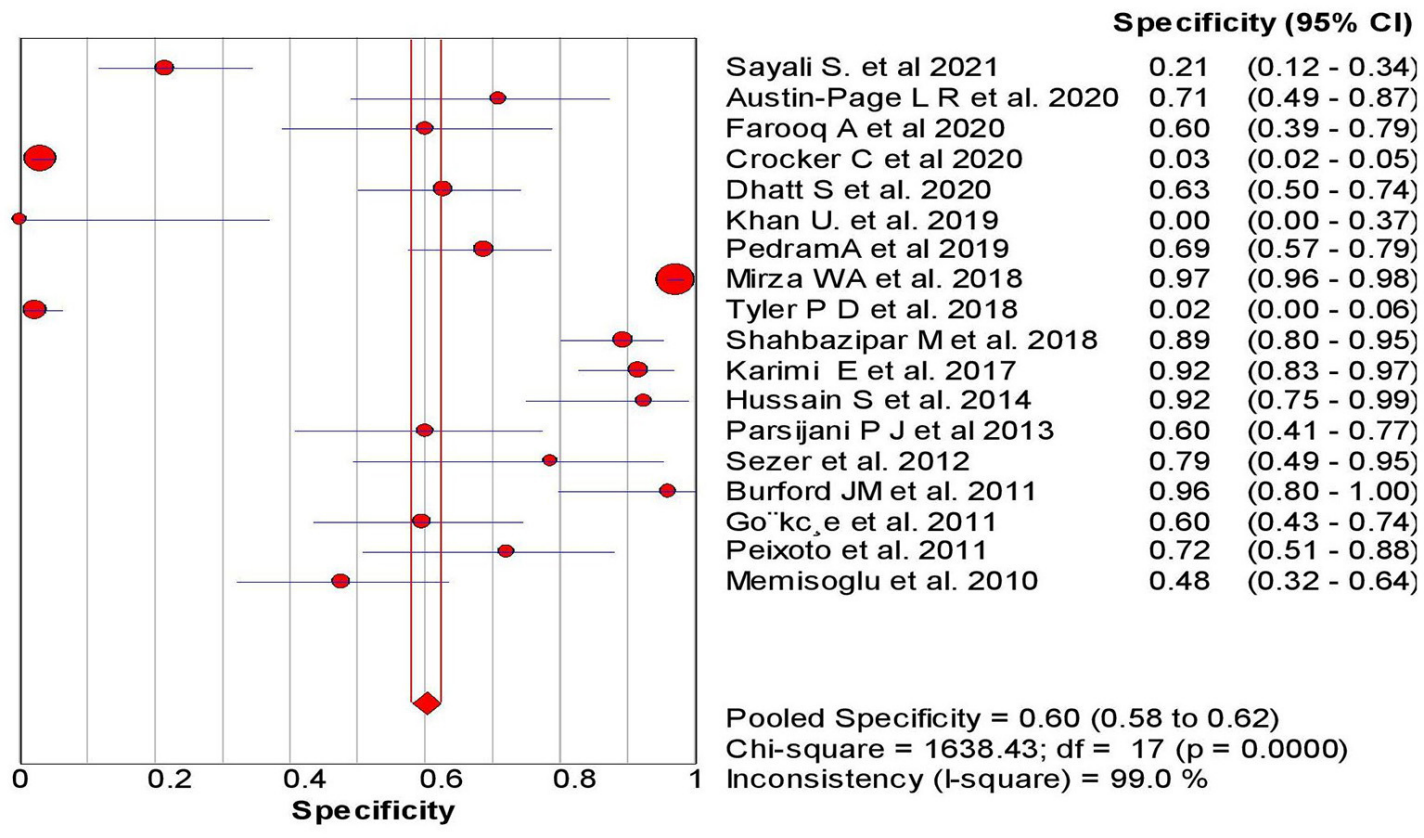
Specificity of ultrasound in acute appendicitis cases.

**Figure 4:**
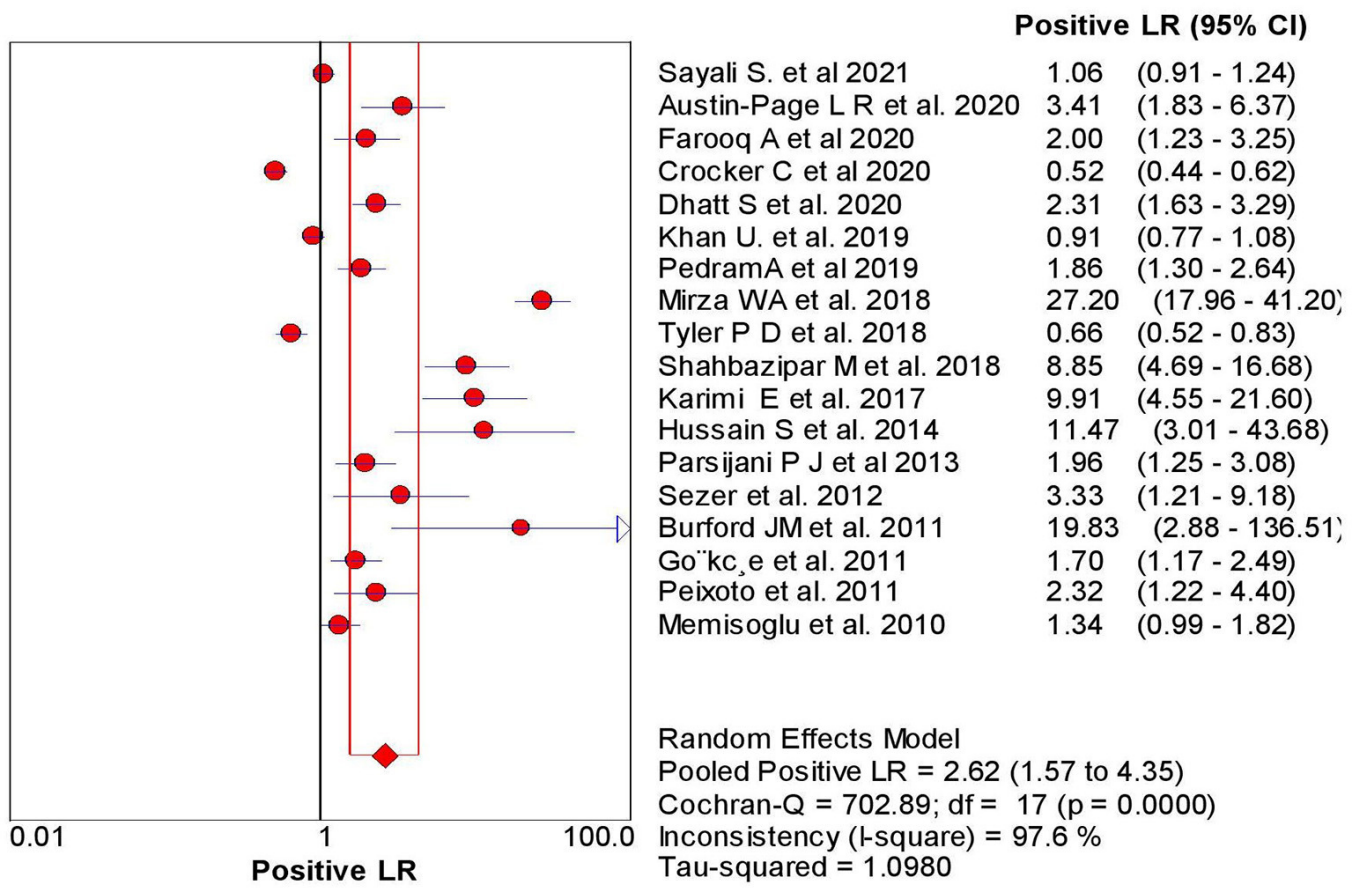
Positive Likelihood ratio of ultrasound in acute appendicitis cases.

**Figure 5:**
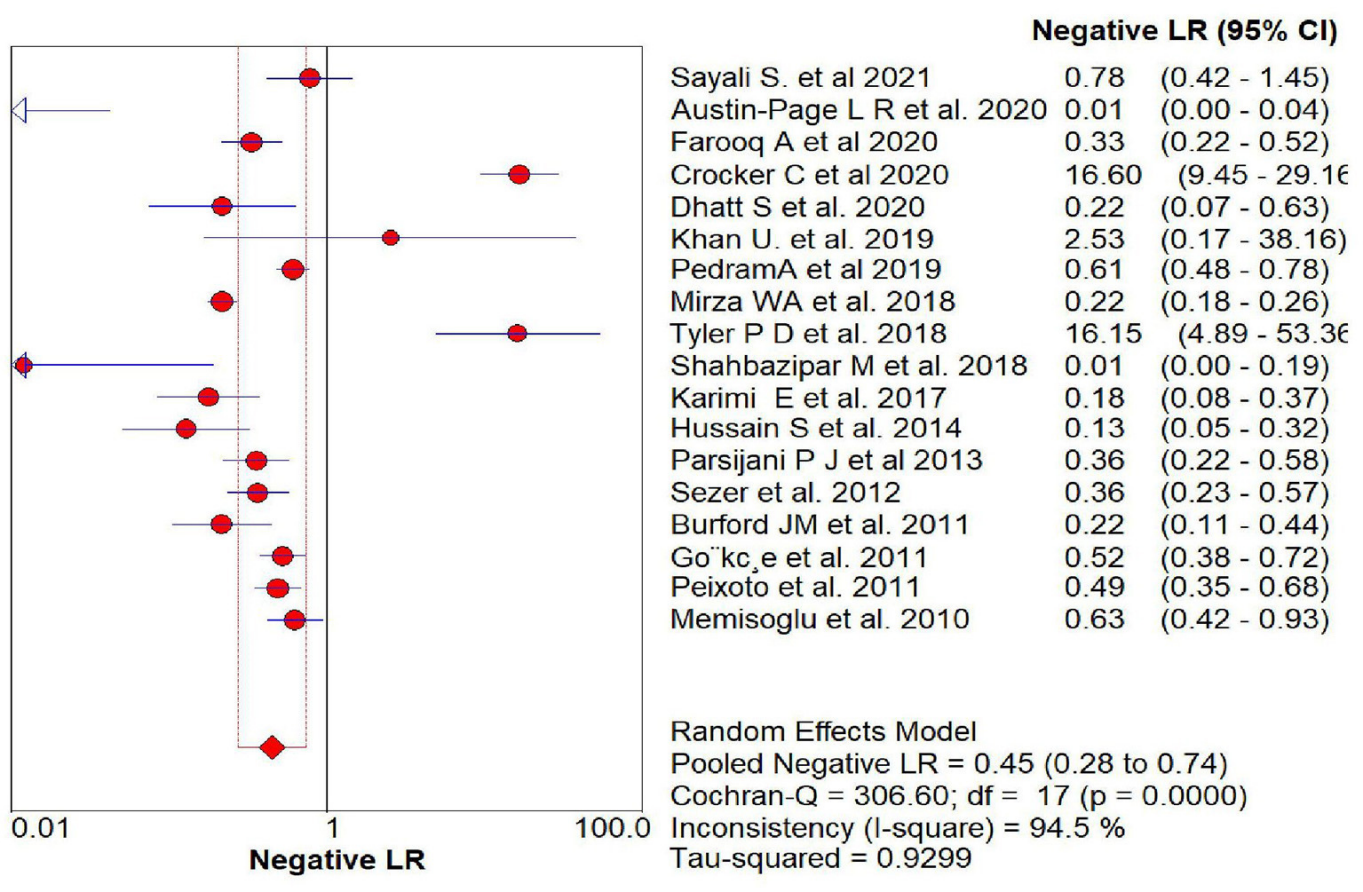
Negative Likelihood ratio of ultrasound in acute appendicitis cases.

**Figure 6:**
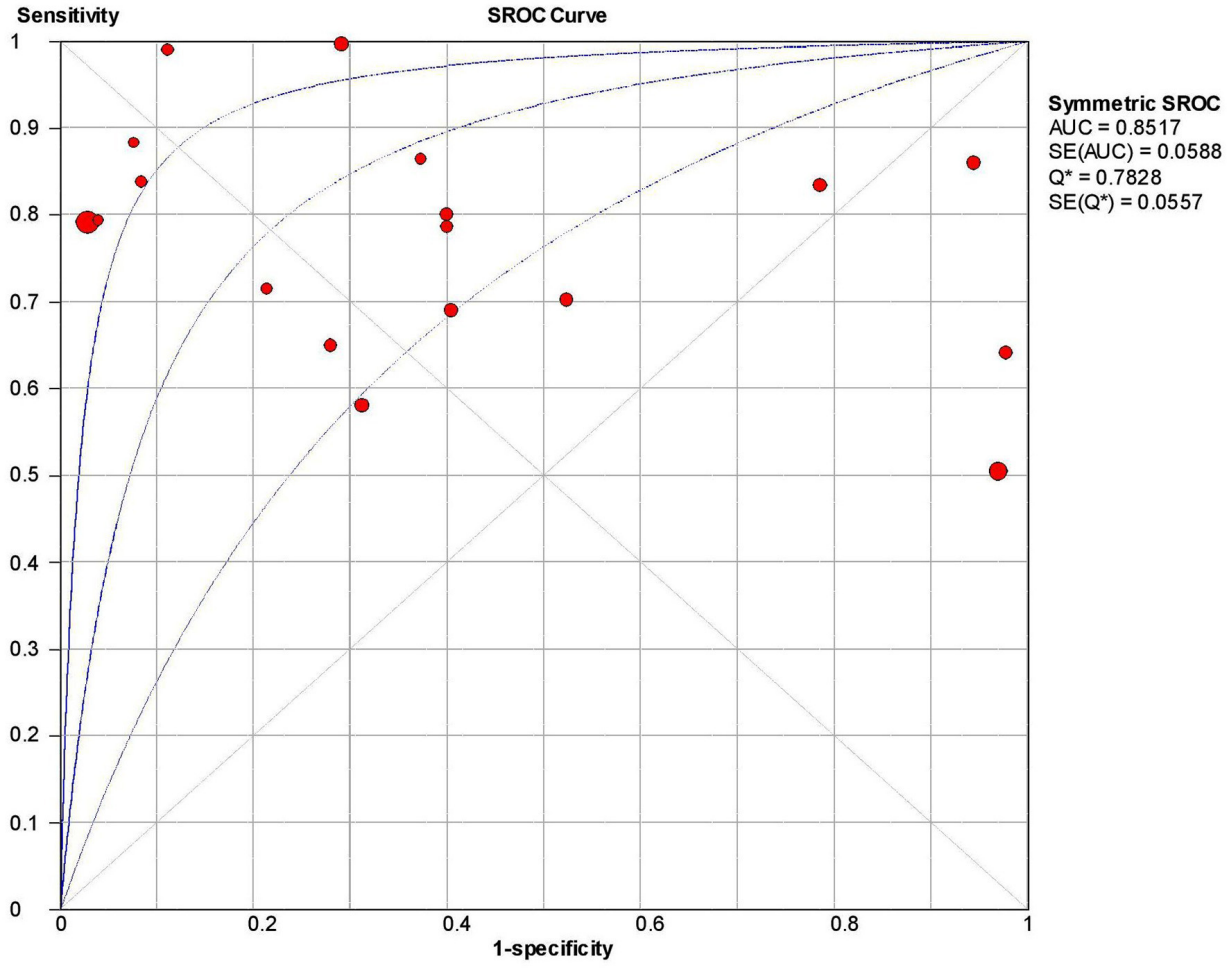
SROC curve of ultrasound in acute appendicitis cases.

**Figure 7:**
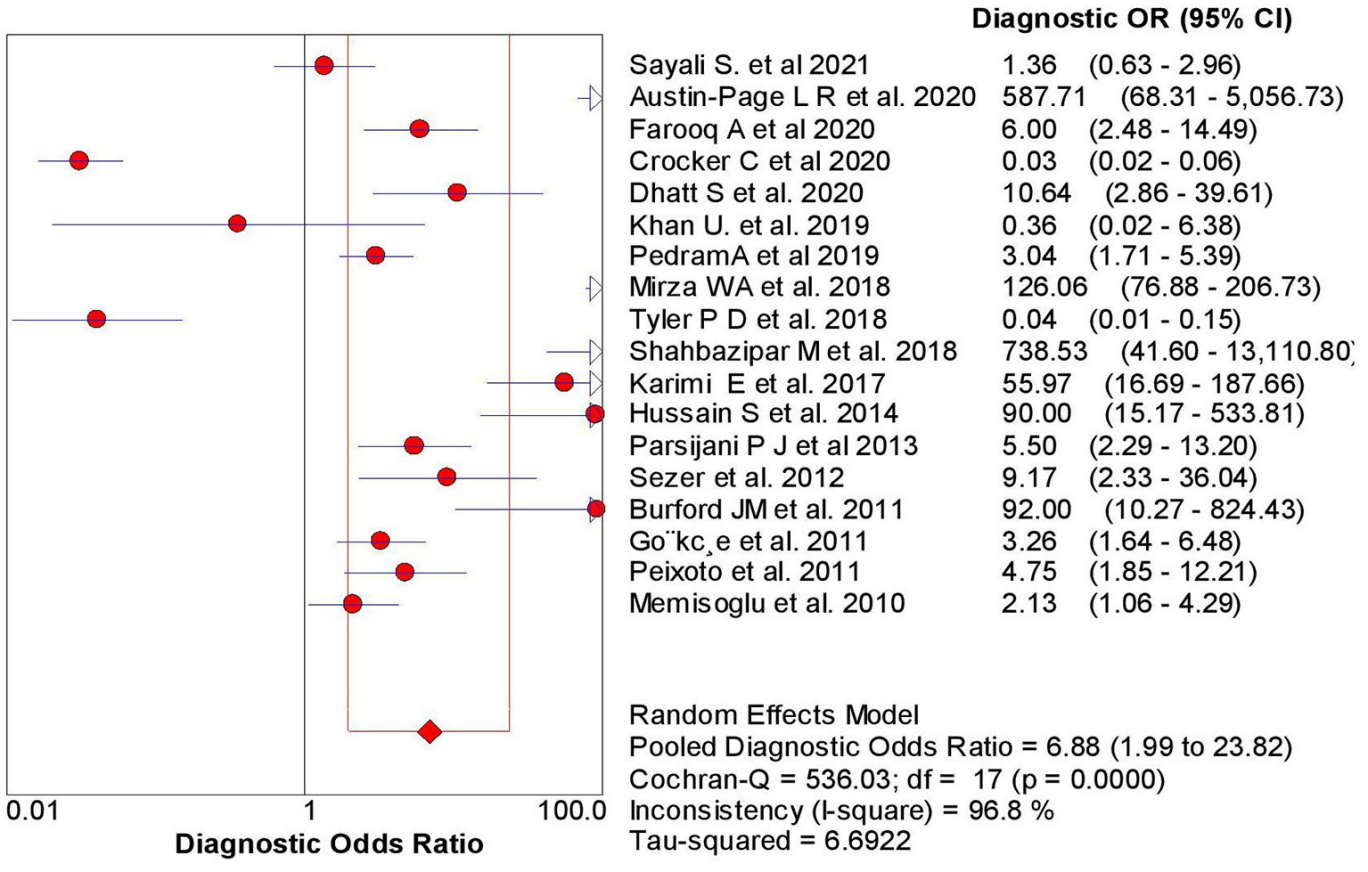
Diagnostic OR of ultrasound in acute appendicitis cases.

The overall sensitivity of the abdominal ultrasound scan in acute appendicitis was 77.2% (95% CI – 75.4–78.9%) when correlated to histopathology, as shown in Figure 2. The overall specificity was 60% (CI – 58–62%), as shown in Figure 3. The overall positive likelihood ratio (PPV) was 2.62 (95% CI-1.57–4.35), as shown in Figure 4 and the overall negative likelihood ratio (NPV) was 0.45 (95% CI-0.28–0.74) as shown in Figure 5. Figure 6 demonstrated an SROC plot showing an estimate of sensitivity vs. specificity and area under the SROC curve. The diagnostic odds ratio was 6.88(1.99–23.82) at 95% CI, demonstrating greater abdominal ultrasound accuracy in diagnosing acute appendicitis, as shown in Figure 7.

The proportion of female participants, number of prospective studies, female participants, type of ultrasound probe, ultrasonographer experience, and clinical probability of acute appendicitis were the covariates that showed statistically significant effects on summary outcomes in the subgroup study (Table 3).

**Table 3:** Exploration of heterogeneity sources; the impact of sample subgroups or participant characteristics on overview sensitivity and specificity.

**Subgroup**	***P*-value**
Full text vs. abstracts	Not available
High vs. low risk of bias	Not available
Prospective vs. retrospective study	0.024^*^
Adults vs. mixed population	0.924
Proportion of female participants	0.05^*^
Proportion of obese participants	Not available
Type of ultrasound probe	0.034^*^
Ultrasonographer experience	0.001^*^
Clinical probability of AA	0.001^*^

*AA, Acute Appendicitis; NA, Not Available; The details could not be retrieved from the report, or only one party was present.*

* *Significant impact of the subgroup on summary results*.

## Discussion

Definitive diagnosis in acute appendicitis has always been challenging because of its non-specific symptoms, signs, and laboratory findings, which can mimic several other pathologies (30). It is considered to be one of the most common abdominal emergency surgeries. However, to avoid the negative appendectomy rate of emergency surgeries, Computed tomography (CT) scan is considered as the gold standard in preoperative diagnosing acute appendicitis patients, and it is seen in the past that preoperative imaging with CT has significantly lowered the negative appendectomy rates (NARs) to 1.7% (31, 32), but it exposes to ionizing radiation, is expensive and time-consuming and has its diagnostic insufficiencies (33).

The present Meta-analysis was an effort to rule out the efficacy of abdominal ultrasound in diagnosing suspected cases of acute appendicitis in all age groups. It can be misdiagnosed, especially in young women, children, and elderly patients. This Meta-analysis was a systematic update from 2010 to 2021, and a total of 18 articles were selected to rule out the sensitivity, specificity, PPV, NPV, and Diagnostic odd ratios. When correlated with histopathology, the present analysis showed an overall sensitivity of 77% with 95% CI varying from 75 to 79% based on the studies included. The studies included a wide range of sensitivity varying from 50 to 100% (95% CI – 41–100%). The present analysis showed an overall specificity of 60%, with 95% CI varying from 58 to 62%. The studies included showed a wide range of sensitivity varying from 0 to 97% (95% CI – 0%−98%); when compared to other previous studies.

Similarly, Doria et al. (33) compared CT and ultrasound in pediatric and adult populations. Again, surgery or follow-up was the gold standard. In the adult population, the combined sensitivity and specificity were 83 and 93%, respectively.

Giljaca et al. (34) showed a sensitivity of 69% and specificity of 81%, which was different from the present study. The present study showed a high sensitivity rate compared to Giljaca et al. (34), stating the ability to identify acute appendicitis patients more accurately. Another similar Meta-analysis was performed by Orr et al. (35), showing sensitivity and specificity of 84.7 and 92.1%; however, the specificity of Orr et al. was very high when compared with the present meta-analysis showing a high ability to identify the patients without acute appendicitis, which differ from the present analysis. Orr et al. (35) concluded that the US should not be used in the diagnosis of acute appendicitis cases where clinical signs and symptoms are definitive. According to Orr et al., ultrasound should be used in cases where patients are with intermediate probability of acute appendicitis after the clinical evaluation.

Another study done by Weston et al. (36) showed high sensitivity and specificity with a value of 88.3 and 92.3%. Still, this study did not take any reference standards compared to the present study; we took histopathology as the gold standard to compare the results of the US to reduce the chances of false-negative rate. The positive likelihood ratio and negative likelihood ratio were 2.62 with a 95% CI of 1.57–4.35 and 0.45 (95% CI – 0.28–0.74%). The diagnostic odds ratio of the present study was 6.88 (95% CI 1.99–23.82), showing a reasonable accuracy rate of abdominal ultrasound in diagnosing acute appendicitis. The SROC curve obtained in the present analysis shows the combined effect of sensitivity and specificity, indicating the inclination of the curve toward the top left depicting good diagnostic accuracy of abdominal ultrasound.

Likewise, only Korean papers were reviewed by Yu et al. (37). Although most of the included participants were checked up on, surgery and histopathology do not seem to be the reference norm. The US had a sensitivity and specificity of 86.7 and 90.0%, respectively.

van Randen et al. (38) specifically compared CT and US; however, surgery was not the reference norm in all patients, and others were followed up without surgery. The US had a sensitivity and accuracy of 78 and 83%, respectively. Carroll et al. (39) compared the sensitivity and specificity of the US performed by surgeons to histopathology or US performed by a radiologist, with sensitivity and specificity of 92 and 96%, respectively.

Only the histopathology record of the surgical specimen served as the reference standard in our research. As a result, our study's sensitivity and accuracy are much more minor than previously reported. This disparity may result from a rigidly enforced standard under which all patients were subjected to surgery. In addition, this fact may lead to an underestimation of sensitivity in our sample because the patient group was more chronically ill.

The limitation of the present study is that the variability in the type of sonographer as skilled and experienced radiologists can reduce the chance of false-negative results. The diagnostic accuracy of the US could be compared with other methods of imaging to see the variability. Analysis of studies showing accuracy based on techniques using Color Doppler to ultrasound examination and various scoring systems based on patient's history, physical examination, and laboratory tests can further improve the diagnostic accuracy rate of ultrasound in acute appendicitis.

## Conclusion

Although imaging with CT has significantly lowered the negative appendectomy rates but still due to its high cost, high ionization radiation exposure risks, and its complexity for interpretation makes ultrasound technique an efficient diagnostic aid mainly in suspected cases of children, young females, and elderly patients. In addition, it is a simple, non-invasive, non-ionizing radiation technique, and its easy availability makes it an effective diagnostic alternative to reduce the rate of unnecessary surgeries in acute appendicitis.

## Data Availability Statement

The raw data supporting the conclusions of this article will be made available by the authors, without undue reservation.

## Author Contributions

JF: concept and designed the study. XZ: analyzed data and drafting of the manuscript. LC: collected the data and helped in data analysis. SL: proofreading and final editing and guarantor of the manuscript. All authors read and approved the final version of the manuscript.

## Conflict of Interest

The authors declare that the research was conducted in the absence of any commercial or financial relationships that could be construed as a potential conflict of interest.

## References

[1] Douglas CD Macpherson NE Davidson PM Gani JS. Randomised controlled trial of ultrasonography in diagnosis of acute appendicitis, incorporating the Alvarado score. BMJ. (2000) 321:919–22. 10.1136/bmj.321.7266.919 11030676 PMC27498

[2] Flum DR Morris A Koepsell T Dellinger EP. Has misdiagnosis of appendicitis decreased over time? A population-based analysis. JAMA. (2001) 286:1748–53. 10.1001/jama.286.14.1748 11594900

[3] Lee SL Walsh AJ Ho HS. Computed tomography and ultrasonography do not improve and may delay the diagnosis and treatment of acute appendicitis. Arch Surg. (2001) 136:556–62. 10.1001/archsurg.136.5.556 11343547

[4] Wilms IM Suykerbuyk-de Hoog DE de Visser DC Janzing HM. Appendectomy versus antibiotic treatment for acute appendicitis. Cochrane Database Syst Rev. (2020) 10:CD008359. 10.1002/14651858.CD008359.pub3 33001448 PMC10631378

[5] Bergeron E. Clinical judgment remains of great value in the diagnosis of acute appendicitis. Can J Surg. (2006) 49:96–100. 16630419 PMC3207532

[6] Lane MJ Liu DM Huynh MD Jeffrey RB Jr Mindelzun RE Katz DS. Suspected acute appendicitis: nonenhanced helical CT in 300 consecutive patients. Radiology. (1999) 213:341–6. 10.1148/radiology.213.2.r99nv44341 10551210

[7] Gwynn LK. The diagnosis of acute appendicitis: clinical assessment versus computed tomography evaluation. J Emerg Med. (2001) 21:119–23. 10.1016/S0736-4679(01)00353-5 11489398

[8] Raman SS Lu DSK Kadell BM Vodopich DJ Sayre J Cryer H. Accuracy of nonfocused helical CT for the diagnosis of acute appendicitis: a 5-year review. AJR Am J Roentgenol. (2002) 178:1319–25. 10.2214/ajr.178.6.1781319 12034591

[9] Lessin MS Chan M Catallozzi M Gilchrist BF Richards C Manera L. Selective use of ultrasonography for acute appendicitis in children. Am J Surg. (1999) 177:193–6. 10.1016/S0002-9610(99)00002-1 10219853

[10] Pedram A Asadian F Roshan N. Diagnostic accuracy of abdominal ultrasonography in pediatric acute appendicitis. Bull Emerg Trauma. (2019) 7:278–83. 10.29252/beat-070311 31392228 PMC6681883

[11] Samudra SS Munde AS. Comparative study between clinical diagnosis using modified Alvarado score and ultrasound imaging in decreasing negative appendectomy rate. Int Surg J. (2021) 8:1185–9. 10.18203/2349-2902.isj20211295

[12] Austin-Page LR Pham PK Elkhunovich M. Evaluating changes in diagnostic accuracy of ultrasound for appendicitis: does practice make perfect? J Emerg Med. (2020) 59:563–72. 10.1016/j.jemermed.2020.06.001 32732135

[13] Farooq A Zameer S Khadim R. Diagnostic accuracy of ultrasound in acute appendicitis in comparison with alvarado score keeping histopathological correlation as gold standard. Pak Armed Forces Med J. (2020) 70:807–11.

[14] Crocker C Akl M Abdolell M Kamali M Costa AF. Ultrasound and CT in the diagnosis of appendicitis: accuracy with consideration of indeterminate examinations according to STARD guidelines. AJR Am J Roentgenol. (2020) 215:639–44. 10.2214/AJR.19.22370 32406773

[15] Dhatt S Sabhaney V Bray H Skarsgard E D. Improving the diagnostic accuracy of appendicitis using a multidisciplinary pathway. J Pediatr Surg. (2020) 55:889–92. 10.1016/j.jpedsurg.2020.01.040 32067806

[16] Khan U Kitar M Krichen I Maazoun K Ali Althobaiti R Khalif M. To determine validity of ultrasound in predicting acute appendicitis among children keeping histopathology as gold standard. Ann Med Surg. (2018) 38:22–7. 10.1016/j.amsu.2018.11.019 30591836 PMC6305696

[17] Mirza WA Naveed MZ Khandwala K. Utility and accuracy of primary and secondary ultrasonographic signs for diagnosing acute appendicitis in pediatric patients. Cureus. (2018) 10:e3779. 10.7759/cureus.3779 30854267 PMC6395012

[18] Tyler PD Carey J Stashko E Levenson RB Shapiro NI Rosen CL. The potential role of ultrasound in the work-up of appendicitis in the emergency department. J Emerg Med. (2019) 56:191–6. 10.1016/j.jemermed.2018.10.034 30594351

[19] Shahbazipar M Seyedhosseini J Vahidi E Motahar Vahedi HS Jahanshir A. Accuracy of ultrasound exam performed by emergency medicine versus radiology residents in the diagnosis of acute appendicitis. Eur J Emerg Med. (2019) 26:272–6. 10.1097/MEJ.0000000000000547 29438133

[20] Karimi E Aminianfar M Zarafshani K Safaie A. The accuracy of emergency physicians in ultrasonographic screening of acute appendicitis; a cross-sectional study. Emerg. (2017) 5:e22. 28286829 PMC5325891

[21] Hussain S Rahman A Abbasi T Aziz T. Diagnostic accuracy of ultrasonography in acute appendicitis. J Ayub Med Coll Abbottabad. (2014) 26:12–17. 25358207

[22] Parsijani P J Zarandi NP Paydar S Abbasi H Bolandparvaz S. Accuracy of ultrasonography in diagnosing acute appendicitis. Bull Emerg Trauma. (2013) 1:158–63. 27162849 PMC4789451

[23] Sezer TO Gulece B Zalluhoglu N Gorgun M Dogan S. Diagnostic value of ultrasonography in appendicitis. Adv Clin Exp Med. (2012) 21:633–6. 23356200

[24] Burford JM Dassinger MS Smith SD. Surgeon-performed ultrasound as a diagnostic tool in appendicitis. J Pediatr Surg. (2011) 46:1115–20. 10.1016/j.jpedsurg.2011.03.040 21683208

[25] Gökçe AH Aren A Gökçe FS Dursun N Barut AY. Akut apandisitte ultrasonografinin güvenilirligi [Reliability of ultrasonography for diagnosing acute appendicitis]. Ulus Travma Acil Cerrahi Derg. (2011) 17:19–22. 10.5505/tjtes.2011.82195 21341129

[26] Peixoto RO Nunes TA Gomes CA. Indices of diagnostic abdominal ultrasonography in acute appendicitis. Influence of gender and physical constitution, time evolution of the disease and experience of radiologist. Rev Col Bras Cir. (2011) 38:105–11. 10.1590/S0100-69912011000200007 21710048

[27] Memisoglu K Karip B Mestan M Onur E. The value of preoperative diagnostic tests in acute appendicitis, retrospective analysis of 196 patients. World J Emerg Surg. (2010) 5:5. 10.1186/1749-7922-5-5 20181221 PMC2834661

[28] Whiting PF Rutjes AW Westwood ME Mallett S Deeks JJ Reitsma JB. QUADAS-2: a revised tool for the quality assessment of diagnostic accuracy studies. Ann Intern Med. (2011) 155:529–36. 10.7326/0003-4819-155-8-201110180-00009 22007046

[29] Review Manager (RevMan) [Computer Program] (2012) Version 5.2. Copenhagen: The Nordic Cochrane Centre, The Cochrane Collaboration.

[30] Becker T Kharbanda A Bachur R. Atypical clinical features of pediatric appendicitis. Acad Emerg Med. (2007) 14:124–9. 10.1197/j.aem.2006.08.009 17192449

[31] Raja AS Wright C Sodickson AD Zane RD Schiff GD Hanson R. Negative appendectomy rate in the era of CT: an 18-year perspective. Radiology. (2010) 256:460–5. 10.1148/radiol.10091570 20529988

[32] Soyer P Dohan A Eveno C Naneix AL Pocard M Pautrat K. Pitfalls and mimickers at 64-section helical CT that cause negative appendectomy: an analysis from 1057 appendectomies. Clin Imaging. (2013) 37:895–901. 10.1016/j.clinimag.2013.05.006 23845254

[33] Doria AS Moineddin R Kellenberger CJ Epelman M Beyene J Schuh S. US or CT for diagnosis of appendicitis in children and adults? A meta-analysis. Radiology. (2006) 241:83–94. 10.1148/radiol.2411050913 16928974

[34] Giljaca V Nadarevic T Poropat G Nadarevic VS Stimac D. Diagnostic accuracy of abdominal ultrasound for diagnosis of acute appendicitis: systematic review and meta-analysis. World J Surg. (2017) 41:693–700. 10.1007/s00268-016-3792-7 27864617

[35] Orr RK Porter D Hartman D. Ultrasonography to evaluate adults for appendicitis: decision making based on meta-analysis and probabilistic reasoning. Acad Emerg Med. (1995) 2:644–50. 10.1111/j.1553-2712.1995.tb03606.x 8521213

[36] Weston AR Jackson TJ Blamey S. Diagnosis of appendicitis in adults by ultrasonography or computed tomography: a systematic review and meta-analysis. Int J Technol Assess Health Care. (2005) 21:368–79. 10.1017/S0266462305050488 16110717

[37] Yu SH Kim CB Park JW Kim MS Radosevich DM. Ultrasonography in the diagnosis of appendicitis: evaluation by meta-analysis. Korean J Radiol. (2005) 6:267–77. 10.3348/kjr.2005.6.4.267 16374085 PMC2684974

[38] van Randen A Bipat S Zwinderman AH Ubbink DT Stoker J Boermeester MA. Acute appendicitis: meta-analysis of diagnostic performance of CT and graded compression US related to prevalence of disease. Radiology. (2008) 249:97–106. 10.1148/radiol.2483071652 18682583

[39] Carroll PJ Gibson D El-Faedy O Dunne C Coffey C Hannigan A. Surgeon-performed ultrasound at the bedside for the detection of appendicitis and gallstones: systematic review and meta-analysis. Am J Surg. (2013) 205:102–8. 10.1016/j.amjsurg.2012.02.017 22748292

